# Retraction: Upregulation of microRNA-340 promotes osteosarcoma cell apoptosis while suppressing proliferation, migration and invasion by inactivating the CTNNB1-mediated Notch signaling pathway

**DOI:** 10.1042/BSR-20171615_RET

**Published:** 2020-09-16

**Keywords:** CTNNB1, Invasion, MicroRNA-340, Notch signaling pathway, Osteosarcoma, Proliferation

This article is being retracted from *Bioscience Reports* at the request of the authors following receipt of a notification from multiple readers, alerting the authors to unusual features in the Western blots of Figure 8B as well as repeated features in Figure 12A. The authors have confirmed that parts of the work were outsourced; however, they have failed to disclose this within the paper or provide details of the collaborator upon request.

The authors are unable to confirm the validity of the results and wish to retract the article. The Editor-in-Chief and Editorial Board agree with the retraction

